# Disentangle-and-aggregate feature learning (DAFNet) for motor bearing fault diagnosis

**DOI:** 10.1038/s41598-025-34490-6

**Published:** 2026-02-05

**Authors:** Jing Tang, Canjun Xiao, Dong Guo, Jiao Bao, Xu Ji, Chenyu Wang

**Affiliations:** 1 Institute of Mine Intelligence, Chengdu Technological University, Chengdu, 610031 China; 2 China Southwest Architectural Design and Research Institute Corp.Ltd, Chengdu, 610041 China

**Keywords:** Motor bearing, Fault diagnosis, DAFNet, Lightweight, Energy science and technology, Engineering, Mathematics and computing

## Abstract

To address the issues of parameter redundancy and low computational efficiency in traditional convolutional neural networks (CNNs) for motor bearing fault diagnosis, which are caused by increasing network depth, this paper proposes a Disentangle-and-Aggregate Feature Learning Network (DAFNet). This method is designed to overcome the challenges of model deployment on resource-constrained edge devices. Moving beyond the conventional strategy of simply stacking layers, DAFNet innovatively adopts a hierarchical disentanglement and aggregation mechanism. It utilizes a secondary splitting strategy to disentangle shallow, medium, and deep features, followed by terminal feature fusion to achieve an effective representation of fault information. Experimental results based on the CWRU dataset demonstrate that DAFNet achieves a 100% average accuracy in fault diagnosis while significantly reducing both computational overhead and parameter count. Compared with existing mainstream lightweight models, this method exhibits superior generalization performance and inference speed, providing new theoretical support for the efficient application of deep learning in industrial embedded systems.

## Introduction

Electric motors, as industrial power cores, require stable operation for production safety. Bearings, critical motor components, transmit loads while maintaining shaft positioning, enduring significant radial/axial stresses. Their condition directly determines motor stability and lifespan. Statistically, bearing failures account for 30% of motor malfunctions^1^ and manifest as wear, fatigue, corrosion, or fractures. These latent faults are challenging to detect early; severe cases trigger motor shutdowns, disrupt production, and pose safety risks. Therefore, advancing bearing fault diagnosis is of great significance to ensuring operational reliability and improving economic efficiency.

Early motor bearing fault detection relied on manual visual inspection of operating conditions and manual auditory monitoring of abnormal sounds. This approach was highly experience-dependent, often detecting faults only when they had progressed to a severe stage. With advances in sensor and signal processing technologies, the engineering field has started using sensors to collect temperature and vibration signals for monitoring motor and bearing operation. In this context, we also apply techniques like filtering and denoising to extract useful information from complex signals. However, signal processing/analysis algorithms and technologies are usually complex, requiring strong mathematical and professional expertise—creating a high threshold for learning and implementation. In recent years, AI advancements have driven fault diagnosis methods towards intelligent evolution^2^. Specifically, these intelligent methods involve collecting, organizing, integrating, and analyzing data to develop an automated decision-making model, with decisions and actions centered on data. Such methods are also known as data-driven methods.

Data-driven methods rely heavily on reliable and valuable data. For instance, Liu^3^ diagnosed bearing faults using bearing images as model inputs. However, this method necessitates an operational halt, leading to production losses and a lack of real-time monitoring capability. Choudhary et al.^4–6^ employed thermal imaging, which can operate under high-speed conditions but requires exposed bearings, making it unsuitable for most industrial applications. Beyond image data, more studies have utilized digital data, primarily including noise^7^, current^8–12^, and vibration^10,13–22,26^ data. In noisy environments, acoustic data is highly susceptible to interference. Current data offers non-intrusive monitoring, but the stator current is predominantly composed of the power supply frequency component. In this case, the characteristic fault frequency of bearings is not prominent in the current spectrum and tends to be easily masked by the fundamental frequency and other harmonics. Consequently, sophisticated filtering is required to extract effective features^23^. In contrast, vibration data can directly reflect motor bearing faults, enable data collection without requiring equipment shutdown, and facilitate real-time monitoring of the motor’s operational status. Therefore, this research focuses on vibration-based diagnostic methods for motor bearing faults.

Data-driven methods primarily fall into two categories: those grounded in traditional machine learning and those based on deep learning. Methods based on traditional machine learning^4,12,16,19,21,22,27^ typically rely on intricate feature extraction, and the selection of these features significantly influences the diagnostic outcomes. With the continuous advancement of artificial intelligence, numerous deep learning-based methods for motor bearing fault diagnostics are emerging. A core advantage of deep learning is its ability to automatically extract features from data, thereby eliminating the manual feature design required by traditional methods^24^. This capability enables the model to achieve relatively optimal diagnostic results by directly processing raw data.

Within the vast array of deep-learning methodologies, CNNs have been extensively applied in fault diagnosis and have achieved notable results. Previous research demonstrated that CNNs can effectively extract valuable features from motor vibration signals^23^. Using vibration signals as inputs, Zhang et al.^14^ enhanced AlexNet for rapid fault identification. In contrast, the study in^17^ employed multi-path convolutions with varying kernel sizes to extract complementary features before classification. Long et al.^26^ improved model generalization through a diversity-regularized snapshot ensemble CNN (ISECNN). Liu et al.^10^ constructed a motor bearing fault diagnosis model based on multi-adversarial domain adaptation. Similarly, Abbasi et al.^15^ and Chang et al.^18^ integrated CNN-LSTM networks to capture both spatial and temporal fault characteristics. Sun et al.^30^ proposed a frequency-adaptive convolutional layer (SCNET), which significantly optimized the performance of Bidirectional Gated Recurrent Units (BiGRU) in fault feature extraction.

Although CNN models have achieved significant progress in fault diagnosis, they are still hindered to some extent by issues such as high structural complexity, excessive parameters, and heavy computational burdens. This potentially leads to slow execution in real-time applications or on resource-constrained devices. On one hand, some researchers transform vibration signals into 2D image data^13–15,25^. While the diagnostic accuracy reaches approximately 99%, this conversion process introduces additional data preprocessing overhead, and 2D CNNs generally exhibit lower parameter efficiency compared to 1D CNNs. On the other hand, existing high-precision models frequently rely on increasing network depth (such as variants of ResNet and DenseNet) to extract richer semantic features. This “layer-stacking” strategy enhances feature extraction capabilities but results in an exponential increase in both parameter count and floating-point operations (FLOPs)^31^. In practical industrial scenarios, embedded monitoring nodes and edge computing devices often face challenges, including limited computational resources, restricted storage space, and stringent real-time requirements^32,33^. Overly large models are not only difficult to deploy, but their high-latency inference also fails to meet the demand for instantaneous motor fault early warning. Currently, several studies have proposed improvements based on network pruning^33^ or lightweight convolutions^34^. For instance, reference^35^ proposed an enhanced lightweight multi-scale CNN that utilizes depthwise separable convolutions and attention mechanisms to reduce computational costs while improving the extraction of weak fault features. However, these methods often come at the expense of sacrificing certain feature extraction capabilities and diagnostic accuracy, making it difficult to strike an ideal balance between “lightweight design” and “high performance.”

Therefore, this paper adopts raw one-dimensional (1D) data as the model input to circumvent the redundant computations associated with 2D conversion and the excessive parameter overhead of 2D CNNs. To address the issue of surging parameter counts caused by deepening network architectures, a motor bearing fault diagnosis method based on DAFNet is proposed. To resolve the aforementioned conflict between resource efficiency and feature representation, DAFNet innovatively introduces a “Disentangle-and-Aggregate” mechanism. Unlike conventional lightweight methods that primarily reduce network depth, our approach employs hierarchical feature disentanglement. This mechanism explicitly separates shallow details from deep semantic information. The aggregation mechanism then synthesizes these critical features through multi-dimensional fusion, ensuring a comprehensive representation. This design enables the model to significantly lower parameter counts and computational costs while maintaining high sensitivity to subtle fault features, thereby facilitating efficient and precise real-time fault diagnosis. The main contributions of this work are summarized as follows:

1. A DAFNet is proposed to address the issue of parameter redundancy in deep models from an architectural perspective. Unlike traditional CNNs that rely on stacking layers for performance gains, DAFNet employs a feature disentanglement strategy to separate feature information at different levels and utilizes a feature reaggregation mechanism to fuse shallow details with deep semantics. This design effectively prevents gradient vanishing and significantly reduces model complexity while ensuring the completeness of feature representation.
2. Realized ultra-fast inference speed and energy-efficient operation for fault diagnosis models in edge computing scenarios. Experimental results demonstrate that DAFNet achieves an inference speed of up to 8,755.51 FPS, which is a 30- to over 140-fold increase compared to mainstream lightweight models such as MobileNetV3-Small and GhostNet. Meanwhile, its computational complexity is as low as 0.1 MFlops, representing only 1/200th to 1/500th of the complexity of competing models. This extreme efficiency advantage eliminates the dependency on high-performance GPUs, providing a breakthrough solution for the intelligent transformation of resource-constrained devices.
3. Experiments on the CWRU benchmark dataset prove the superior accuracy-efficiency balance of the proposed model. Although existing methods generally achieve high accuracy, DAFNet overcomes the bottleneck where high precision inherently requires large parameter volumes. While achieving 100% diagnostic accuracy—matching that of complex deep architectures—DAFNet reduces the model footprint to between 1/38 and 1/91 of conventional lightweight models. This demonstrates that optimal generalization performance can be sustained even within an ultra-compact structure.

## Data preprocessing methods

To meet the input requirements of deep learning models such as Convolutional Neural Networks (CNNs), the raw one-dimensional (1D) vibration signals are reshaped into a single-channel format. This format preserves the time-domain structure of the signal while satisfying the requirements of typical deep learning frameworks for two-dimensional (2D) input tensors (e.g., [Channel, Length]).

An overlapping sliding window technique is employed to segment the long-sequence signals into training samples of a fixed length. Defining the length of each training sample as *L* sampling points and the overlap rate as *ρ*, the sliding step size *S* is calculated as follows:

1 $$S = L \times \left( {1 - \rho } \right)$$

Since the stride must be an integer number of sampling points, *S* is rounded down to the nearest integer. Let the original vibration signal *X* be a time-domain sequence of length* N*:

2 $$X = \, \left[ {x_{1} ,x_{2} , \, ...,x_{N} } \right]$$

The set of generated samples is given by:

3 $$D = \{ d_{0} ,d_{1} , \ldots ,d_{M - 1} \}$$

The total number of samples *M* is given by Eq. (4):

4 $$M=\left(\frac{N-L}{S}\right)+1$$

The samples are stacked to form the sample matrix:

5 $$D=\left[\begin{array}{c}{{\boldsymbol{d}}}_{0}\\ {{\boldsymbol{d}}}_{1}\\ \vdots \\ {{\boldsymbol{d}}}_{M-1}\end{array}\right]= \left[ \begin{array}{c}\begin{array}{c}\begin{array}{ccc}{x}_{1}& {x}_{2}& \dots \\ {x}_{S+1} & {x}_{S+2}& \dots \\ {x}_{2S+1} & {x}_{2S+2}& \dots \end{array} \begin{array}{c}{x}_{L}\\ {x}_{S+L}\\ {x}_{2S+L}\end{array}\\ \end{array}\\ \begin{array}{cc}\vdots & \vdots \\ {x}_{(M-1)S+1}& {x}_{(M-1)S+2}\end{array} \begin{array}{cc}\ddots & \vdots \\ \dots & {x}_{(M-1)S+L}\end{array}\end{array}\right]$$

## DAFNet network

**Fig. 1:**
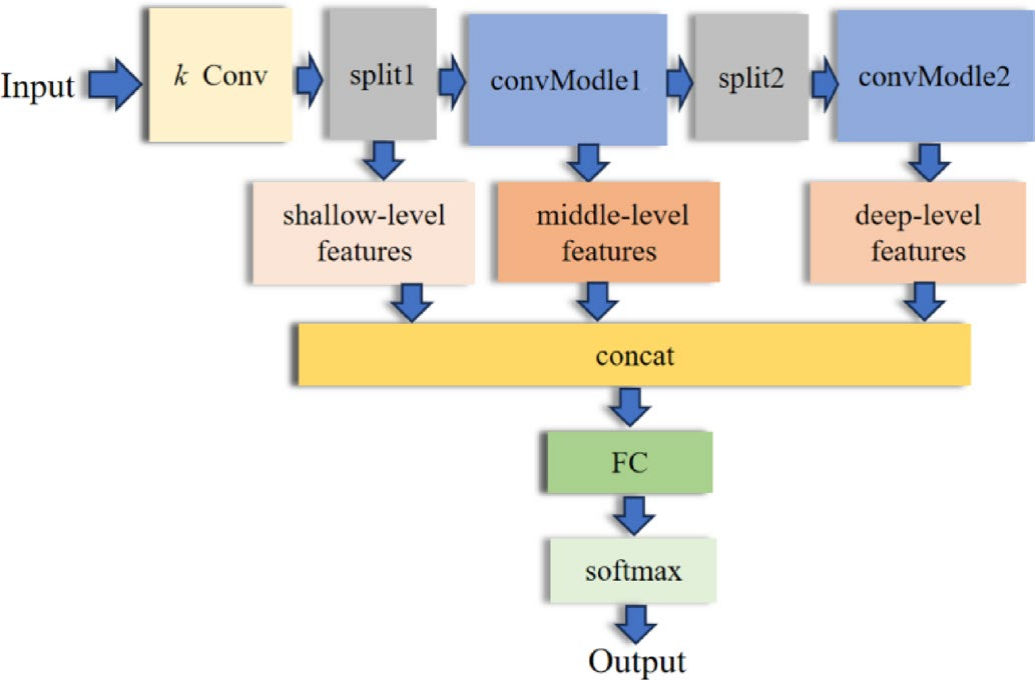
Workflow diagram of DAFNet.

**Fig. 2:**
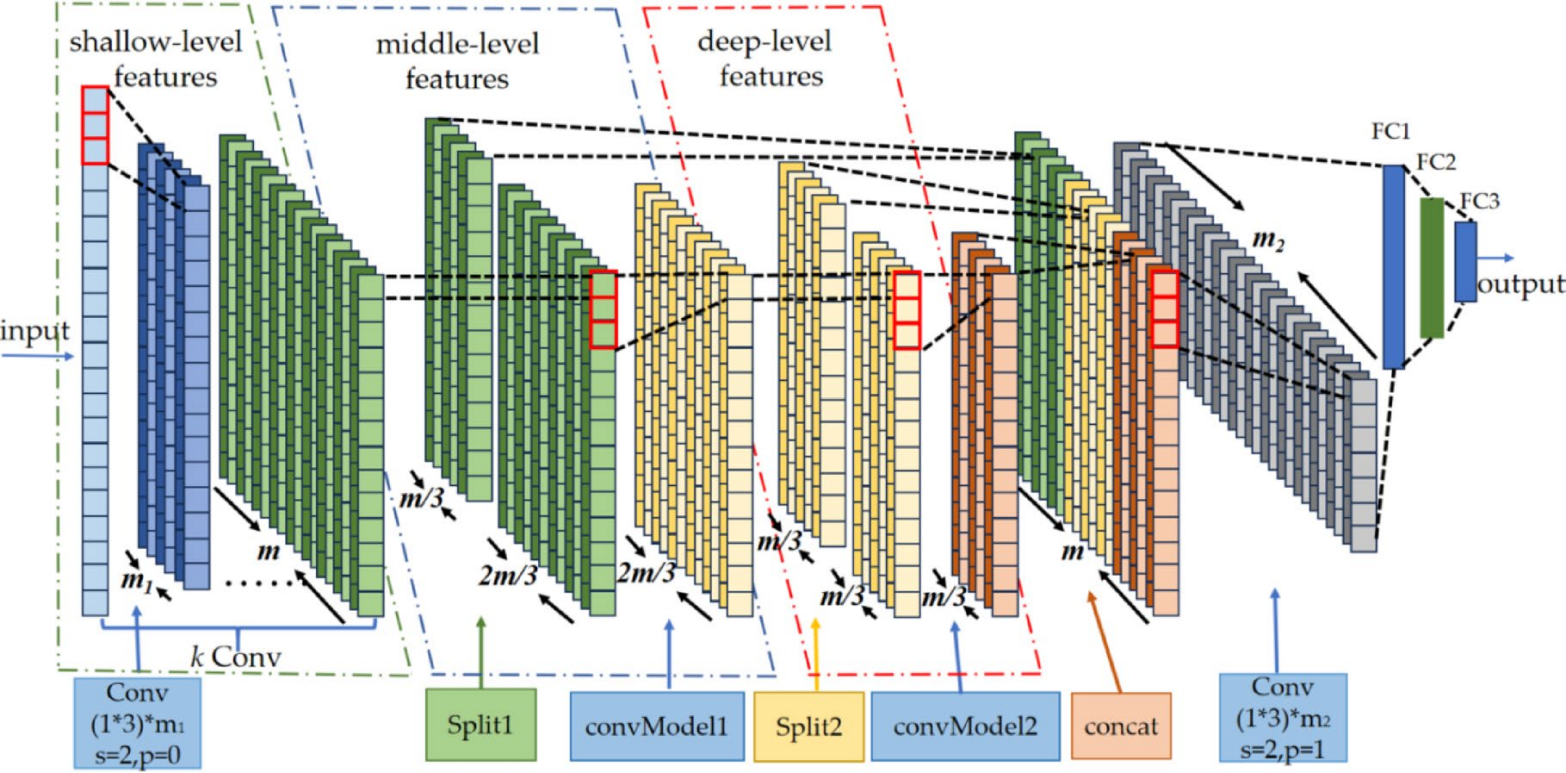
Architecture of the DAFNet.

A classical CNN is composed of multiple convolutional layers, pooling layers, and fully connected layers. As the network’s depth increases, the representational capacity of the CNN gradually improves. Nevertheless, when the network architecture becomes deeper, the number of parameters surges, and so do the training and computational costs. It causes the model to run slowly in real-time applications or on resource-constrained devices. Conversely, if the network architecture is overly shallow, it will be incapable of extracting more abundant deep-level features, resulting in suboptimal model performance. To resolve this contradiction, this paper presents a DAFNet-based motor bearing fault diagnosis method. The workflow of the constructed DAFNet is shown in Fig. 1. Specifically, the architecture of the DAFNet is shown in Fig. 2.

The model first extracts shallow features from the input data using *k* convolutional layers. It then proceeds to the convModel layer to derive middle and deep-level features. A partitioning operation at the Split1 layer disentangles the shallow features and reduces dimensionality by retaining them in one-third of the data, while directing the rest to the convModel1 layer for middle-level feature extraction. The Split2 layer further divides the data to disentangle middle-level features and reduces dimensionality again. One-third of this split retains the middle-level features, and the remaining one-third is passed through the convModel2 layer to extract deep-level features. The disentangled features remain relatively independent and free from mutual interference. Finally, the decoupled shallow, middle, and deep features are concatenated and aggregated. It enables the model to capture both local details, contextual information, and the overall structure of the data simultaneously. The integrated features then pass through one convolutional layer and three fully-connected layers. Subsequently, a softmax classifier is adopted for classification to output the final diagnostic results. In the figure, s (stride) denotes the step size of the convolutional kernel’s movement, and p (padding) represents the number of layers of additional zeros added around the input data.

**Fig. 3:**
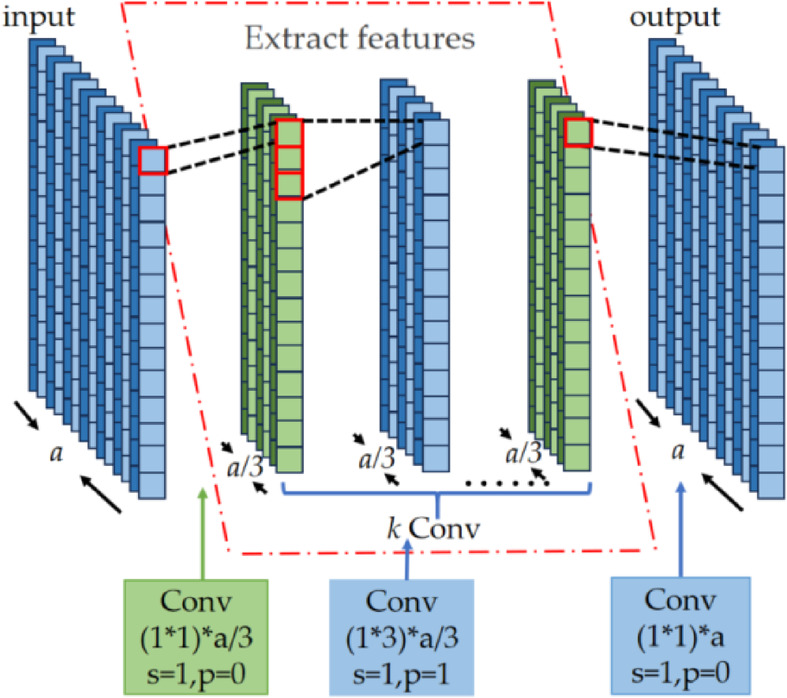
Network architecture of convModel.

In Fig. 2, the network architecture of convModel is shown in Fig. 3. The first convolutional layer employs 1 × 1 convolutional kernels to reduce the data dimensionality. This operation can alleviate the computational load of the subsequent convolutional layers. In the intermediate *k* convolutional layers, the convolution operations adopt 3 × 3 convolution kernels for feature extraction, thereby augmenting the feature representation capacity. Finally, another 1 × 1 convolutional layer restores the data dimensionality for utilization in the next layer. In the figure, *a* denotes the number of input feature channels for the network. Depending on the location of the convMoele module within Fig. 2, the value of a can be 2* m*/3 or *m*/3. And s (stride) denotes the step size of the convolutional kernel’s movement, and p (padding) represents the number of layers of additional zeros added around the input data.

**Fig. 4:**
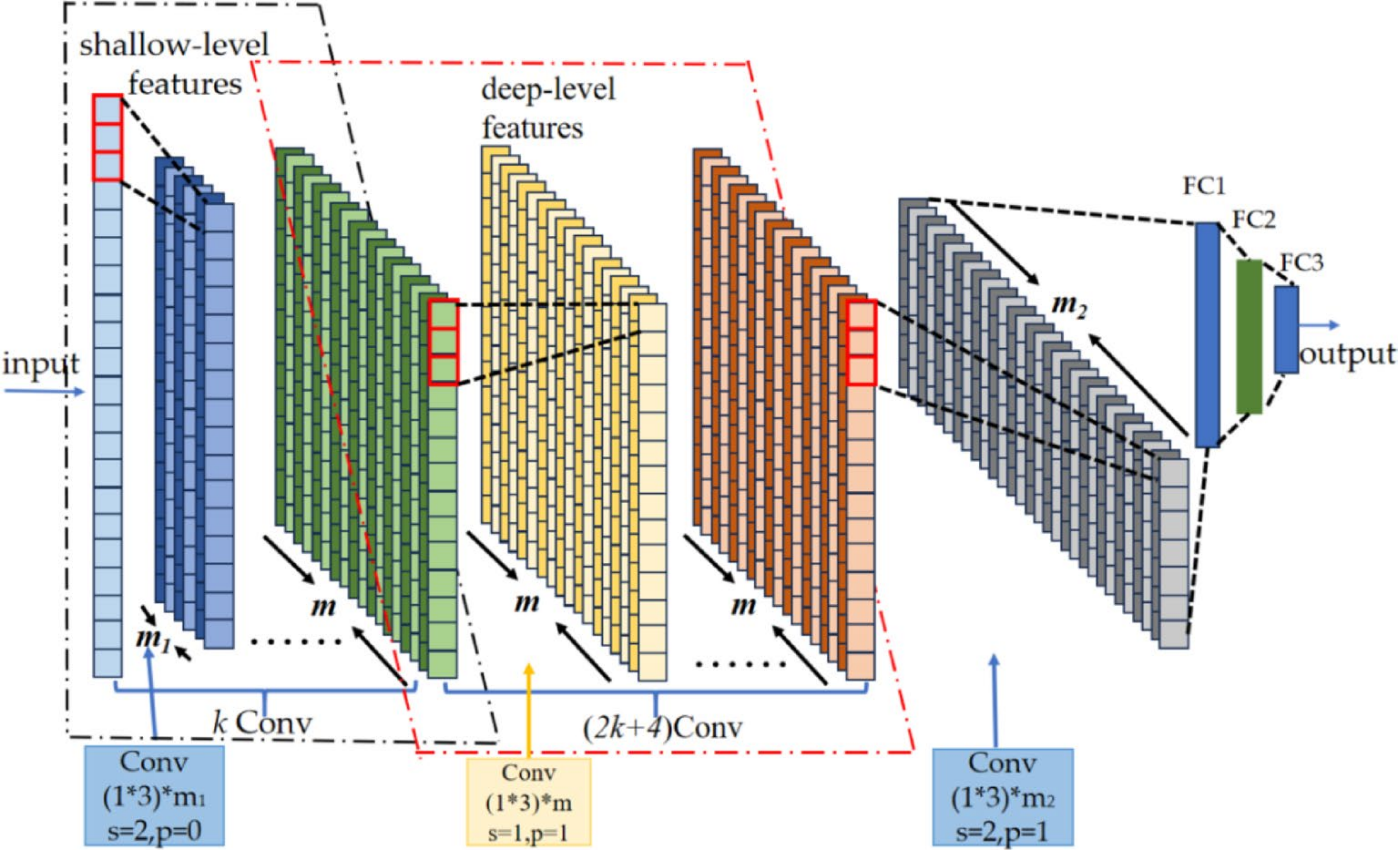
Baseline CNN.

As is evident from Figs. 2 and 3, the DAFNet incorporates a total of 3* k* + 5 convolutional layers for feature extraction. For a more comprehensive comparison with DAFNet, this study constructs a baseline CNN network that also employs 3* k* + 5 convolutional layers for feature extraction. As shown in Fig. 4, the segment dedicated to extracting shallow features, the final convolutional layer, and the fully-connected layers are identical to those of DAFNet. Furthermore, during the feature extraction process within the middle 2* k* + 4 convolutional layers, the data dimension remains unaltered.

As is apparent from Figs. 2 and 4, the disparity between the constructed baseline CNN network and the DAFNet network resides in the components responsible for extracting middle-level and deep-level features. To conduct a comparison of the number of parameters of the two networks, it suffices to compare this specific component. The comparison outcomes are shown in Tables 1 and 2.

**Table 1:** Parameters of baseline CNN.

Layer	Kernel size	Channel num	Kernel num	Parameters
(2* k* + 4) Conv	1 × 3	*m*	*m*	(2* k* + 4) × [*m*(3 × *m* + 1)] = (2* k* + 4) × (3*m*^2^ + *m*)
Total				(2* k* + 4) × (3*m*^2^ + *m*)

**Table 2:** Parameters of DAFNet.

	Layer	Kernel Size	Channel Num	Kernel Num	Parameters
convModel1	1 Conv	1 × 1	2* m/*3	2* m/*9	(2* m*/9) × [1 × (2* m*/3) + 1] = 4*m*^2^/27 + 2* m*/9
	*k* Conv	1 × 3	2* m/*9	2* m/*9	*k*{(2* m*/9) × [3 × (2* m*/9) + 1]} = *k*(4*m*^2^/27 + 2* m*/9)
	1 Conv	1 × 1	2* m/*9	2* m/*3	(2* m*/3) × [1 × (2* m*/9) + 1] = 4*m*^2^/27 + 2* m*/3
convModel2	1 Conv	1 × 1	*m/*3	*m/*9	(*m*/9) × [1 × (*m*/3) + 1] = *m*^2^/27 + *m*/9
	*k* Conv	1 × 3	*m/*9	*m/*9	*k*{(*m*/9) × [3 × (*m*/9) + 1]} = *k*(*m*^2^/27 + *m*/9)
	1 Conv	1 × 1	*m/*9	*m/*3	(*m*/3) × [1 × (*m*/9) + 1] = *m*^2^/27 + *m*/3
Total					[(5* k* + 6) × *m*^2^]/27 + [(*k* + *2*) × *m*]/3

The calculation formula for the number of parameters in a convolutional layer is: Number = Kernel Size × (Width of Kernel × Channel Num + 1).

As is discernible from Tables 1 and 2, the parameter count of the baseline CNN network is:

6 $${\mathrm{Num}}1\, = \,\left( {2 k\, + \,4} \right)\, \times \,\left( {3m^{2} \, + \,m} \right)$$

The number of network parameters of the DAFNet network is:

7 $$\mathrm{Num}2=\frac{(5k+6)\times {m}^{2}}{27}+\frac{(k+2)\times m}{3}$$

**Fig. 5:**
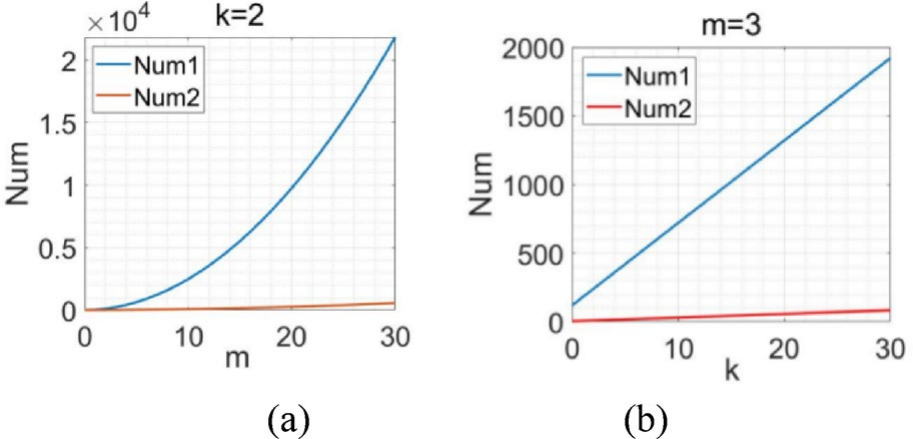
The variation of the number of parameters with respect to *m* and *k*.

The inference drawn from Formula (6) and Formula (7) reveals that with *k* kept invariant and *m* increasing, Fig. 5(a) presents the changing trends of Num1 and Num2. Similarly, with *m* kept invariant and *k* increasing, Fig. 5(b) depicts the changing trends of Num1 and Num2.

As shown in Fig. 5, with the augmentation of the convolutional layer number *k* and the data dimension *m*, the rate of growth of the number of parameters of the baseline CNN network is substantially greater than that of DAFNet. Analysis reveals that the feature disentanglement operation and 1 × 1 convolution kernels in DAFNet enable effective reduction of data dimension, leading to a decrease in the model’s parameter count. Simultaneously, the feature disentanglement operation generates non-interfering shallow, middle, and deep-level features. By fusing these three levels of features, the model captures more comprehensive feature information, thereby enhancing its generalization ability. This approach not only ensures that the number of parameters does not surge significantly but also guarantees that the network can extract deep-layer features. It effectively resolves the aforementioned contradiction.

## Experiments and analysis of experimental results

To comprehensively evaluate the performance and underlying mechanisms of the proposed DAFNet model, a series of experiments was conducted based on the CWRU bearing dataset. The experimental framework is divided into three primary components. First, through decoupled feature visualization, we intuitively demonstrate how DAFNet isolates shallow, intermediate, and deep fault features while elucidating their unique roles in feature extraction. Second, model efficiency comparison experiments were performed to assess the diagnostic accuracy, inference speed, and resource consumption of DAFNet under single working conditions, highlighting its significant advantages in lightweight design. Finally, cross-condition transferability experiments were carried out to address the typical industrial challenge of load variations, verifying the model’s adaptability and generalization performance under different load conditions.

### Data preparation

**Fig. 6:**
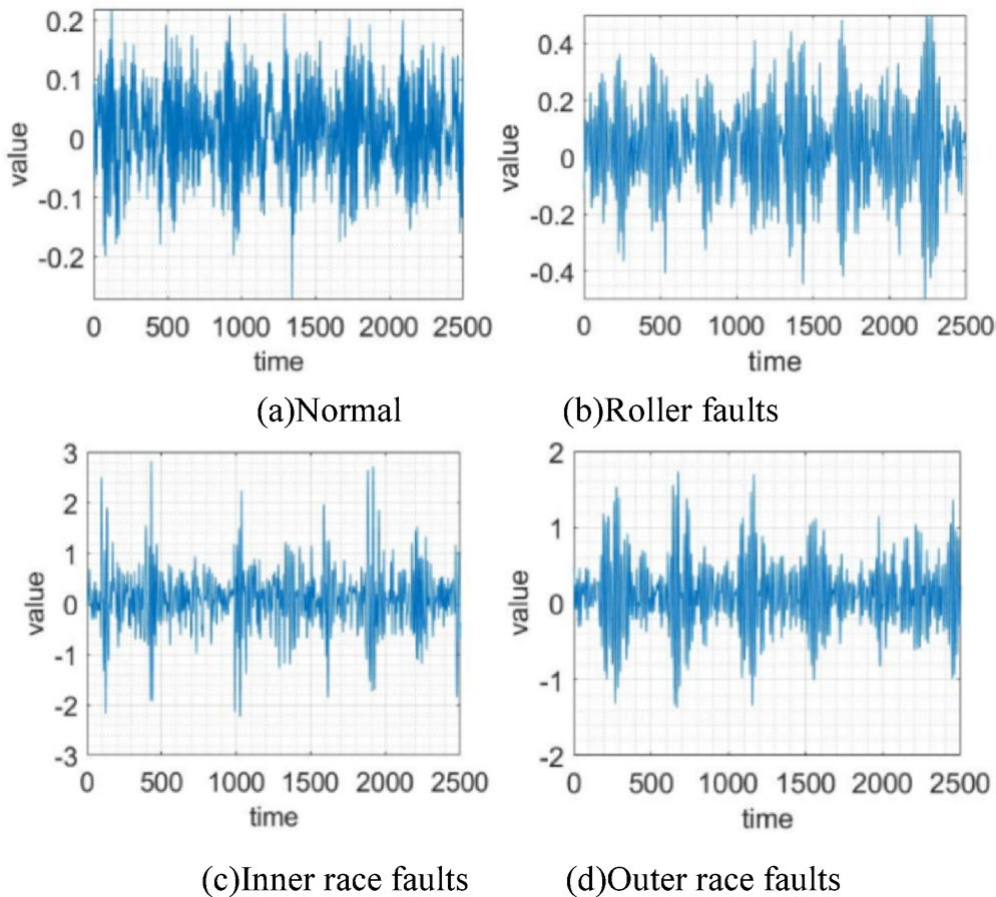
Original vibration signals.

In the experiments, the public dataset from the CWRU rolling bearing test rig is relied on as the experimental data. This paper selects the drive-end data with a sampling frequency of 12 kHz. The motor speeds/loads are 1797 rpm/0 HP, 1772 rpm/1 HP, 1750 rpm/2 HP, and 1730 rpm/3 HP, representing four working conditions designated as W0, W1, W2, and W3, respectively. Each working condition incorporates samples representing four states of the bearing: normal state, roller faults, inner raceway faults, and outer raceway faults. Taking working condition W0 as an example, Fig. 6 shows the original vibration signals of samples for the four bearing states.

According to Eqs. (1) ~ (5), each raw sample is preprocessed. In the experiments, the parameters are set to*ρ* = 0.3 and *L* = 1024. The processed samples are stored independently in NumPy binary format (.npy), with each file containing a single sample of shape (1, *L*). Approximately 1,000 samples are generated for each working condition and are partitioned into training and testing sets at a ratio of 7:3; specifically, 70% of the data is used for model training, while the remaining 30% serves as the test set to verify model performance. Following the split, the training set is denoted as {***d***_1_, ***d***_2_, …, ***d***_*i*_} and the test set as {***d***_*i*+1_, ***d***_*i*+2_, …, ***d***_M-1_}. This partitioning strategy ensures the temporal continuity of the samples and avoids potential temporal information leakage that may result from random splitting.

### Training configuration and hyperparameter settings

This study conducts all experiments on a unified hardware platform with the Windows 10 operating system to ensure fairness. The system configuration includes: an Intel® UHD Graphics GPU (128 MB dedicated memory), an Intel® Core™ i5-10210U processor (base clock speed: 1.60 GHz, maximum turbo frequency: 2.11 GHz), 8.00 GB of system RAM, and a 477 GB TOSHIBA KXG60ZNV512G solid-state drive (SSD). This study uses the deep learning framework in the PyCharm integrated development environment, with Python 3.11 as the implementation language. To ensure the reproducibility of experimental results and effectively evaluate model performance, a consistent training strategy was implemented across all experiments in this study. Random seeds were fixed for network weight initialization and data shuffling to eliminate the influence of stochasticity on the experimental outcomes. The specific training hyperparameter configurations are as follows:

1. Optimizer and Learning Rate Strategy.

The optimization of network parameters is performed using the Stochastic Gradient Descent (SGD) algorithm. Compared to adaptive optimizers, SGD often demonstrates superior generalization performance in certain non-convex optimization landscapes. In this study, the momentum factor is set to 0.2 to accelerate convergence and suppress oscillations. The initial learning rate is fixed at 0.001. Throughout the 500 training epochs illustrated in Fig. 11, a constant learning rate strategy is maintained, without the introduction of weight decay or learning rate warm-up mechanisms.

(2) Batch Size and Loss Function.

The training process adopts a mini-batch input strategy, with the batch size fixed at 32. As this is a classification task, Cross-Entropy Loss is selected as the objective function to quantify the discrepancy between the predicted probability distribution of the model output and the ground truth labels. Its mathematical formulation is as follows:

8 $$L=-\frac{1}{N}\sum_{i=1}^{N}\sum_{c=1}^{C}{y}_{i,c}log({p}_{i,c})$$

where *N* denotes the number of samples in each batch (i.e., 32), *C* represents the total number of classes, *y*_*i,c*_ is the ground truth label (0 or 1) indicating whether sample *i* belongs to class *c*, and *p*_*i,c*_ signifies the predicted probability for class *c* of sample *i*.

### Visualization and analysis of decoupled features

**Fig. 7:**
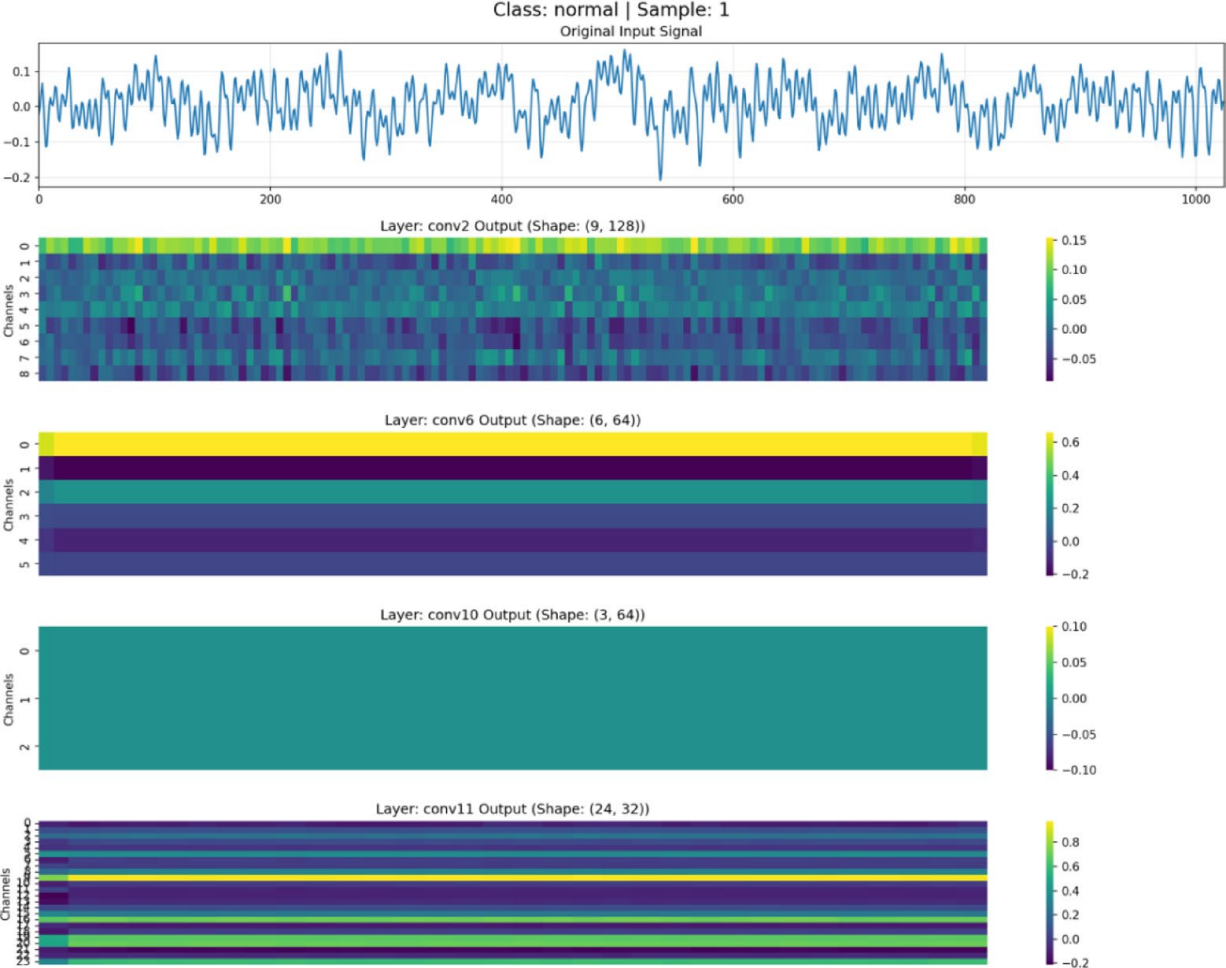
Feature visualization under normal state.

**Fig. 8:**
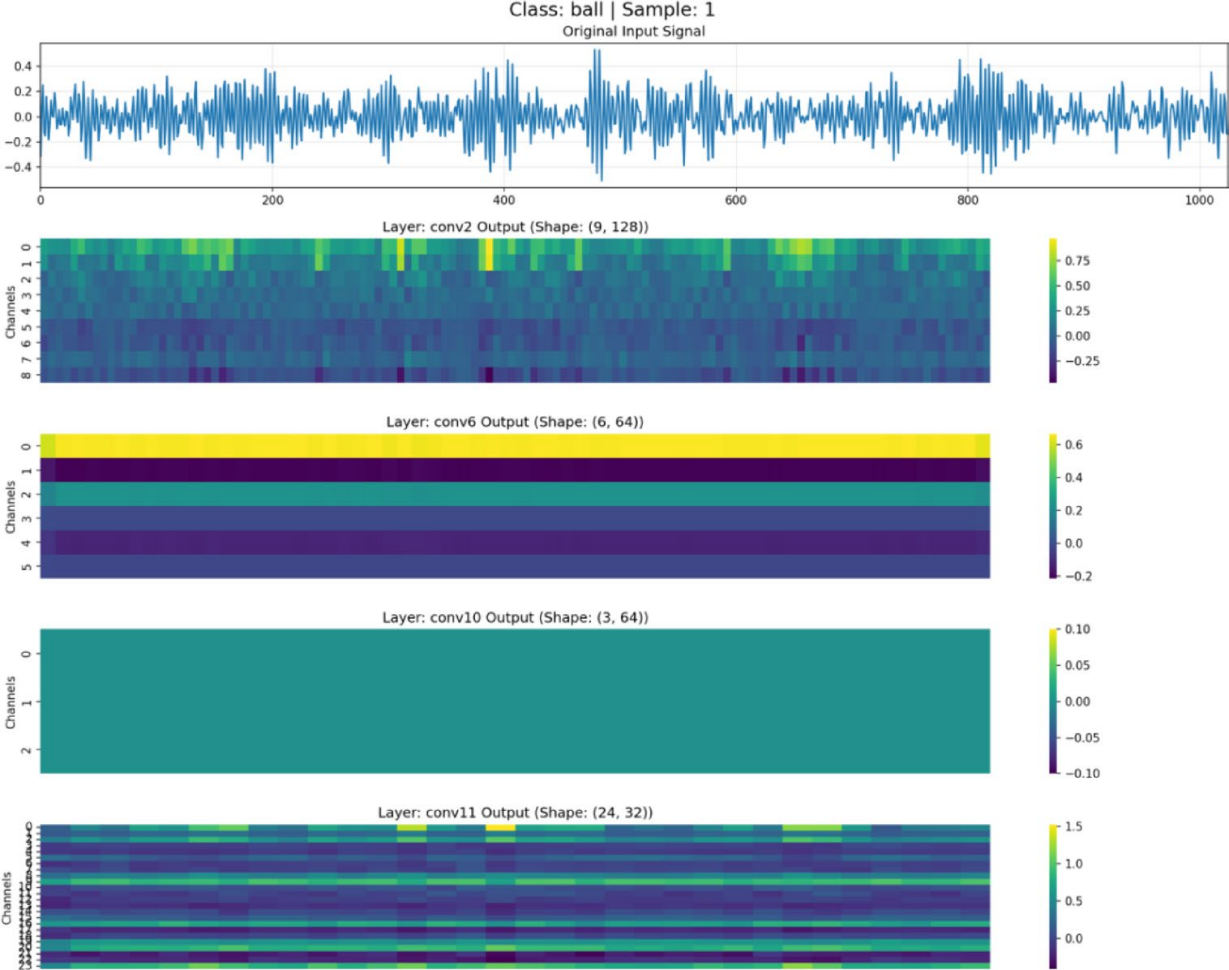
Feature visualization of roller fault.

**Fig. 9:**
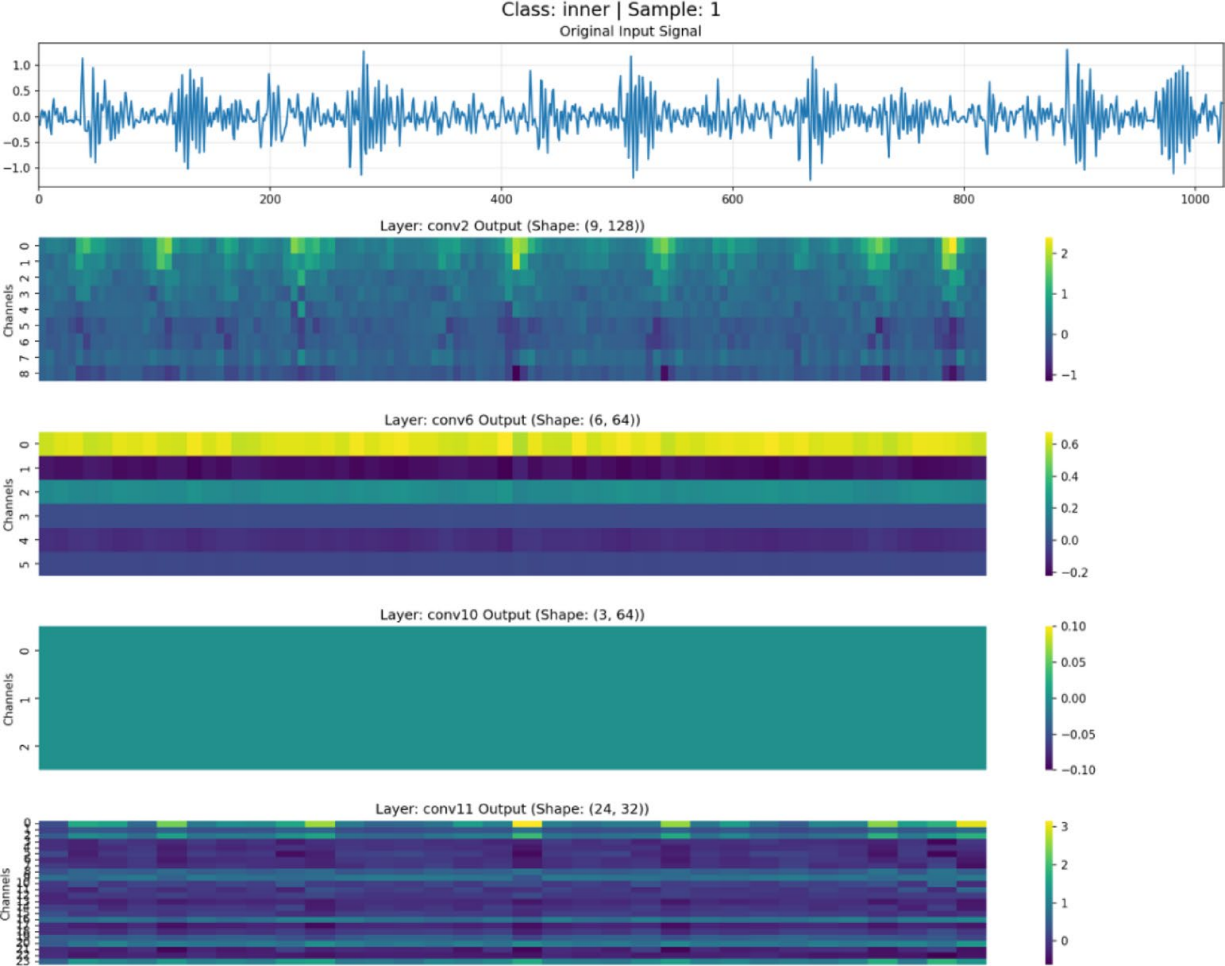
Feature visualization under inner race fault.

**Fig. 10:**
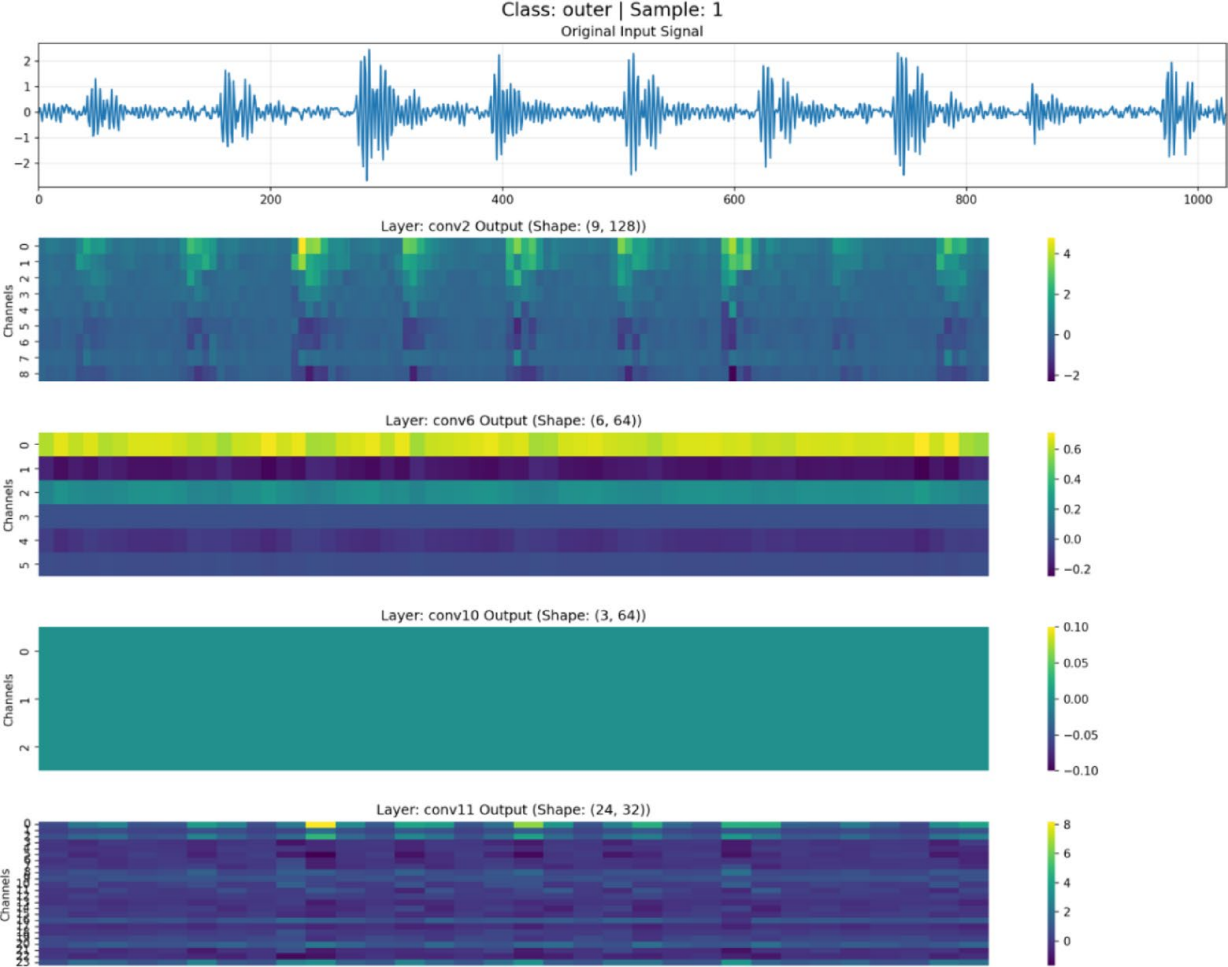
Feature visualization under outer race fault.

To validate the effectiveness of the feature disentanglement mechanism in DAFNet, feature maps at different depths were visualized and analyzed, as illustrated in Figs. 7, 8, 9 and 10. These figures display the raw signals under the normal state and three distinct fault conditions, along with the corresponding feature responses from the shallow (Conv2), middle(Conv6), deep (Conv10), and aggregation (Conv11) layers.

As illustrated in Figs. 7, 8, 9 and 10, the characteristics of the features at different levels are analyzed as follows:

*Shallow Features (Conv2)*: As shown in the Conv2 maps, shallow features exhibit high spatial resolution. In the outer and inner race fault samples, the feature heatmaps display distinct vertical stripes that precisely align with the temporal positions of transient impulses in the raw vibration signals. This indicates that the shallow branch primarily acts as a filter, preserving high-frequency details and time-domain localization of fault shocks, although some background noise remains.

*Middle Features (Conv6)*: As the network depth increases, the resolution of the feature maps in Conv6 decreases. The network begins to suppress background noise and focuses on structural information. The heatmaps reveal clear periodic activation patterns, suggesting that the intermediate branch is responsible for extracting the periodicity and local contextual information of fault signals, which is crucial for distinguishing faults with different frequency characteristics.

*Deep Features (Conv10)*: The feature maps of Conv10 are further abstracted; they no longer contain specific waveform details but instead manifest as global activations across the channel dimension. This represents highly abstract global semantic information. The deep branch exhibits strong translation invariance, providing the discriminative features necessary for the final classification decision.

*Aggregated Features (Conv11)*: The final Conv11 layer fuses the features from the three levels mentioned above. The visualization results demonstrate that these aggregated features retain both the fine-grained details from the shallow layers and the abstract semantics from the deep layers. Through this aggregation mechanism, DAFNet leverages multi-view information for diagnosis, compensating for the limitations of single-depth features and thereby achieving high-precision fault diagnosis.

The significant morphological disparities among feature maps at different hierarchical levels intuitively validate the efficacy of the disentanglement strategy in DAFNet, as evidenced by the following three aspects:

1. Physical Separation of Details and Semantics: Shallow features (Conv2) consist primarily of high-frequency vertical stripes that align precisely with time-domain impulses, whereas deep features (Conv10) manifest as low-frequency global activation patches. The remarkably low similarity in texture and morphology between these two levels indicates that fine-grained details are “frozen” at the front-end and are not forcibly smoothed into the deeper layers. This effectively achieves the physical separation of detail information from semantic information.
2. Noise Isolation and Feature Purity: While shallow feature maps retain substantial background noise, the intermediate and deep feature maps rapidly transition to smooth and structured representations. This sharp discontinuity in noise levels suggests that non-fault-related background noise is isolated within the shallow branches. This prevents noise from interfering with the extraction of deep semantic features, thereby ensuring the purity of the high-level representations.
3. Independent Evolution of Multi-source Features: The feature maps in the fusion layer (Conv11) clearly exhibit the concatenation of features from different hierarchies. It is possible to explicitly distinguish which channels provide detailed textures and which provide categorical semantics. The coexistence of these multi-source features demonstrates that features at each level evolve independently without mutual interference, thus successfully verifying the “disentanglement” design.

### Model efficiency comparative analysis

Model efficiency is pivotal in determining whether a model can be deployed on resource-constrained edge devices. To satisfy the feature disentanglement rules of DAFNet, as illustrated in Figs. 2, the number of feature channels following the shallow feature extraction stage is *m*. These features are subsequently split in a 1:2 ratio before entering the intermediate feature extraction stage; therefore, m must be a multiple of three to ensure proportional partitioning across the shallow, intermediate, and deep feature sets. Here, *k* denotes the number of convolutional layers and must be a positive integer. Given that smaller values of *k* and *m* directly result in a lower parameter count, we conducted exhaustive experiments covering all combinations of *k* ∈ {1,2,3} and* m* ∈ {3,6,9,12,15,18}. The results indicate that the configuration with *k* = 2 and *m* = 9 strikes an ideal balance between diagnostic precision and parameter efficiency. The final parameter counts for DAFNet are detailed in Table 3.

**Table 3:** Parameters of the DAFNet network constructed in this paper.

Layer	Kernel Size	Channel Num	Kernel Num	Parameters
Conv	1 × 3	1	6	24
Conv	1 × 3	6	9	171
convModel1	Refer to Table 2			78
convModel2				
Conv	1 × 3	9	24	672
FC				41,129
Total				42,078

In this section, we conduct comparative experiments with six well-known lightweight networks, including SqueezeNet, ShuffleNet, MicroNet, and a baseline CNN. The network performance is evaluated based on metrics including the number of parameters, FPS, MFlops, and accuracy, with the specific comparison results shown in Table 4.

**Table 4:** Comparison of model efficiency.

Network	Parameters	FPS	MFlops	Accuracy
ShuffleNet	228,844	136.75	11.82	100%
SqueezeNet	354,372	569.19	27.62	100%
MicroNet	323,620	62.79	8.45	100%
MobileNetV3-Small	1,615,452	60.66	19.7	100%
GhostNet	3,855,548	282.43	50.63	100%
CNN	44.012	1319	0.22	100%
DAFNet	42,078	8755.51	0.1	100%

As shown in Table 4, although all compared models achieved 100% testing accuracy, the proposed DAFNet demonstrates overwhelming superiority in other metrics. Regarding model scale and complexity, DAFNet achieves extreme compression with a parameter count of only 42,000 and a computational cost as low as 0.1 MFlops. Its parameter count is merely ~ 1/91 of GhostNet and ~ 1/38 of MobileNetV3-Small, while also being significantly lower than ShuffleNet, SqueezeNet, and MicroNet, and even superior to the simple-structured baseline CNN. This reflects exceptional efficiency in storage and energy consumption. The most significant advantage lies in inference speed, where DAFNet achieves a frame rate of 8,755 FPS. This is not only over 140 times faster than MobileNetV3-Small and MicroNet, but also 31 times faster than GhostNet, 15 times faster than SqueezeNet, and 6.6 times faster than the baseline CNN. These results prove that for processing 1D signals, a concise architecture offers far greater real-time advantages than complex lightweight modules, signifying that DAFNet fully meets the rigorous requirements of high real-time industrial scenarios.

**Fig. 11:**
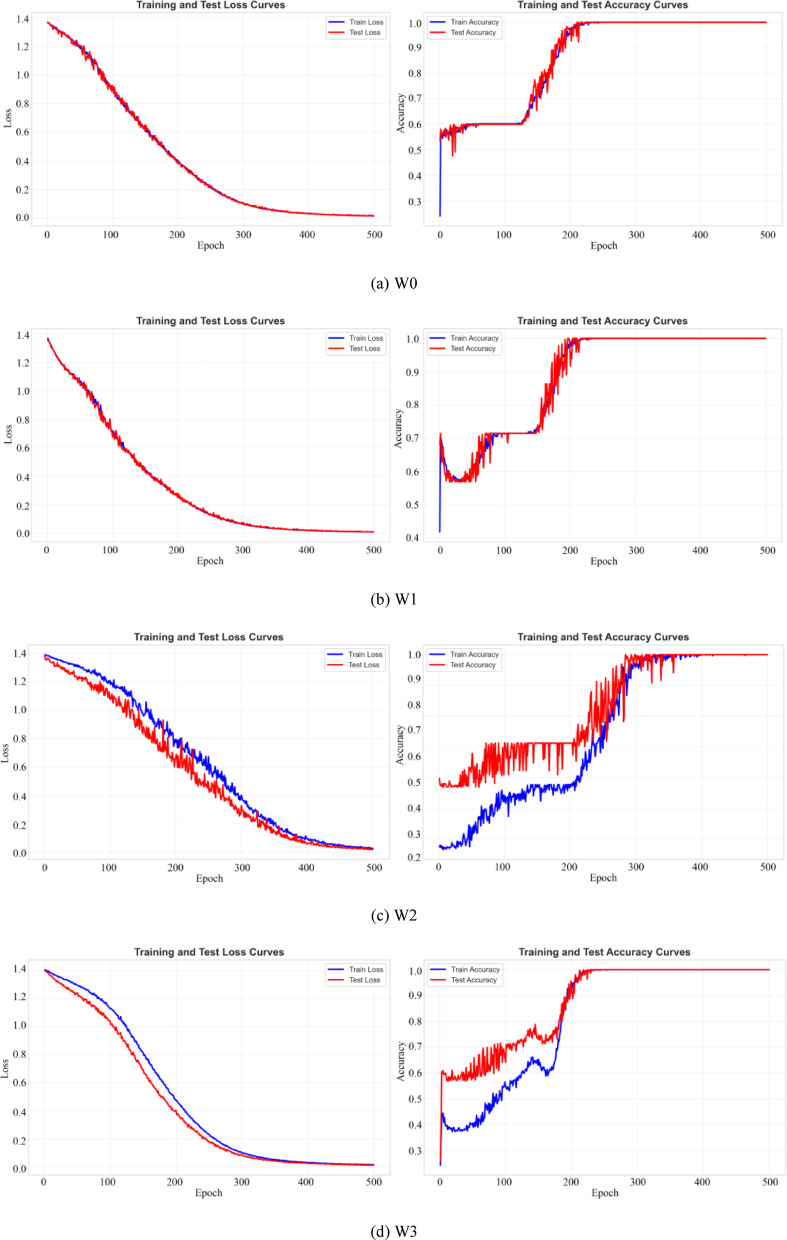
Training process curves.

Figure 11 shows that under the four working conditions, the training loss eventually reaches a small stable value, and the validation loss follows the training loss downward before stabilizing at a level close to the training loss. Meanwhile, the training accuracy climbs to 100%, with the validation accuracy tracking it closely.

To quantitatively assess the disparity with outstanding CNN architectures proposed by other scholars, this paper performs a comprehensive comparison between this network and five others in terms of parameter counts and average accuracy rates. Table 5 shows the comparative results.

**Table 5:** Comparison of the number of parameters and accuracy rates of different CNN networks.

Network	Parameters	Accuracy
Improved AlexNet^14^	947,232	97.95%
ISECNN^26^	10,263,998	99.67%
CNN-LSTM^15^	851,713	98.58%
Improvement of ShufflenetV2^29^	621,128	97.42%
Lightweight CNN^28^	530,000	99.95%
DAFNet	42,078	100%

As shown in Table 5, DAFNet achieves leading performance in both accuracy and lightweight design. With only 42,078 parameters, it attains 100% accuracy, outperforming methods such as ISECNN (99.67%). While the model size is only about 1/12 that of Lightweight CNN—representing a nearly 93% reduction in parameters—it achieves a 2.58 percentage point improvement in accuracy over the Improvement of ShufflenetV2. DAFNet breaks the trade-off between accuracy and complexity, delivering optimal performance with an ultra-compact architecture, which demonstrates its high efficiency in fault diagnosis tasks.

In conclusion, while maintaining high classification accuracy, DAFNet has successfully achieved fewer parameters, faster speed, and lower computational consumption, demonstrating the excellence of its network structure design in terms of computational efficiency.

### Cross-condition generalization capability analysis

In practical industrial environments, equipment operating conditions (such as load) often change, leading to subsequent shifts in data distribution. Therefore, an effective fault diagnosis model must possess strong cross-condition generalization capability. To comprehensively evaluate the model’s generalization capability, this study conducted transfer experiments across various working conditions and performed an integrated assessment using three key metrics: Accuracy, Recall, and F1-Score. The definitions of the evaluation indicators can be found in Eqs. (8) ~ (11).

1. Accuracy:

8 $$A = \frac{TP + TN}{{TP + TN + FP + FN}}$$

2) Precision:

9 $$P=\frac{TP}{TP+FP}$$

3) Recall:

10 $$R=\frac{TP}{TP+FN}$$

4) F1-score:

11 $$F1=\frac{2(P\times R)}{P+R}$$

Among them, TP (True Positive) refers to true positives—specifically, positive cases correctly identified as positive. FP (False Positive) denotes the number of false positives, referring to the quantity of negative classes erroneously predicted as positive classes. TN (True Negative) refers to true negatives—specifically, negative cases accurately identified as negative. FN (False Negative) denotes the number of false negatives, referring to the quantity of positive classes erroneously predicted as negative classes. Accuracy represents the proportion of correctly classified samples among all samples, evaluating the quality of the results. The recall represents the proportion of positive samples that are actually classified as positive among all actual positive samples, assessing the completeness of the results. The F1-score is a result that comprehensively takes into account both precision and recall.

**Fig. 12:**
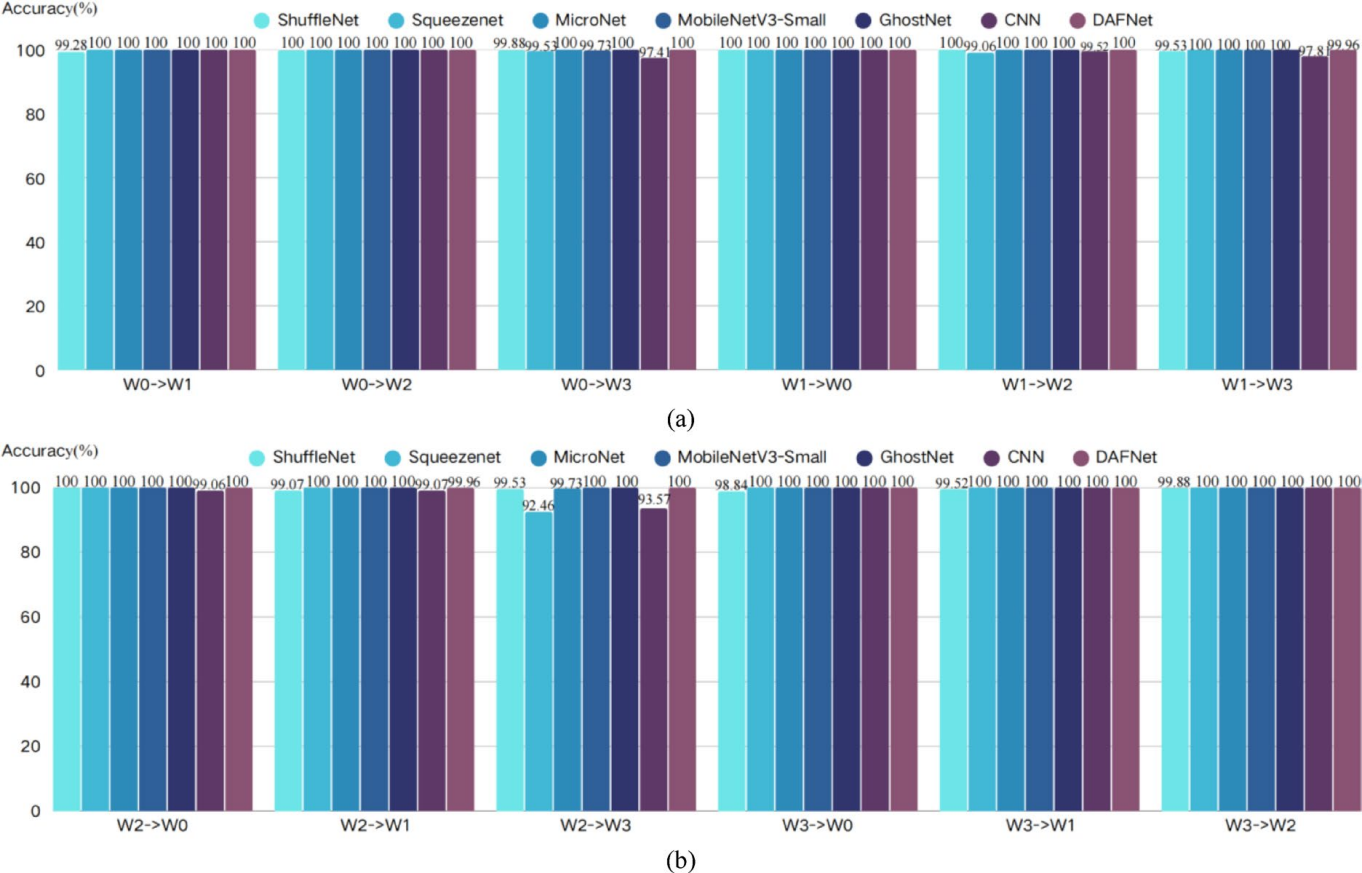
Accuracy visualization results.

**Fig. 13:**
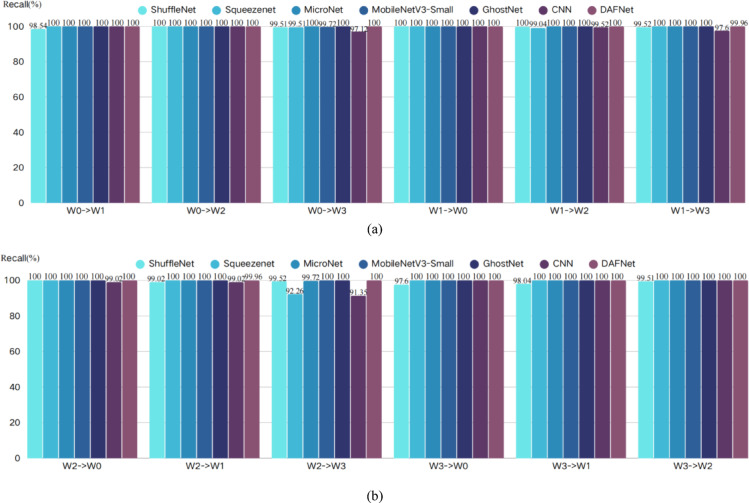
Recall visualization results.

**Fig. 14:**
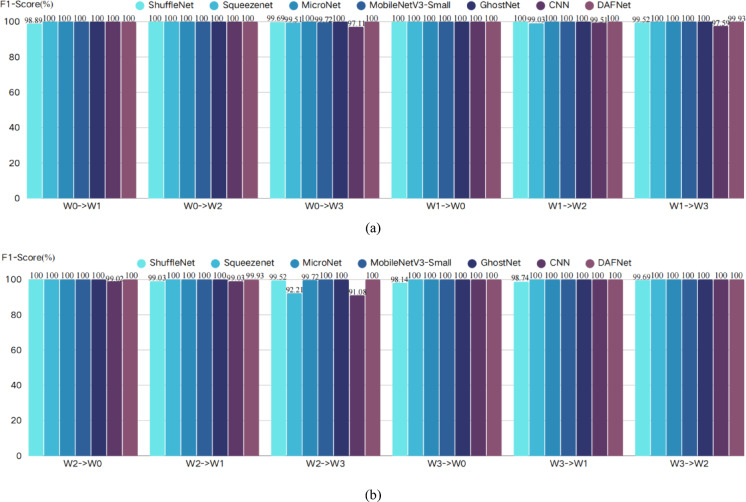
F1-Score visualization results.

To eliminate the potential impact of stochasticity arising from the random initialization of neural network weights, a rigorous statistical evaluation method was adopted. In this study, models trained under one working condition were tested using data from the other three conditions; for instance, “W0—> W1” denotes that a model trained on condition W0 was used to evaluate data from condition W1. For each transfer task, 10 independent trials were conducted using different random seeds. The final results are reported as mean values to comprehensively reflect the performance fluctuations of the model. The key statistical results for this section are summarized in Tables 6, 7 and 8, and the corresponding visualizations are provided in Figs. 12, 13 and 14.

**Table 6:** Accuracy.

Accuracy(%)	W0- > W1	W0- > W2	W0- > W3	W1- > W0	W1- > W2	W1- > W3	W2- > W0	W2- > W1	W2- > W3	W3- > W0	W3- > W1	W3- > W2
ShuffleNet	99.28	100	99.88	100	100	99.53	100	99.07	99.53	98.84	99.52	99.88
Squeezenet	100	100	99.53	100	99.06	100	100	100	92.46	100	100	100
MicroNet	100	100	100	100	100	100	100	100	99.73	100	100	100
MobileNetV3-Small	100	100	99.73	100	100	100	100	100	100	100	100	100
GhostNet	100	100	100	100	100	100	100	100	100	100	100	100
CNN	100	100	97.41	100	99.52	97.81	99.06	99.07	93.57	100	100	100
DAFNet	100	100	100	100	100	99.96	100	99.96	100	100	100	100

**Table 7:** Recall.

Recall(%)	W0- > W1	W0- > W2	W0- > W3	W1- > W0	W1- > W2	W1- > W3	W2- > W0	W2- > W1	W2- > W3	W3- > W0	W3- > W1	W3- > W2
ShuffleNet	98.54	100	99.51	100	100	99.52	100	99.02	99.52	97.60	98.04	99.51
Squeezenet	100	100	99.51	100	99.04	100	100	100	92.26	100	100	100
MicroNet	100	100	100	100	100	100	100	100	99.72	100	100	100
MobileNetV3-Small	100	100	99.72	100	100	100	100	100	100	100	100	100
GhostNet	100	100	100	100	100	100	100	100	100	100	100	100
CNN	100	100	97.12	100	99.52	97.60	99.02	99.02	91.35	100	100	100
DAFNet	100	100	100	100	100	99.96	100	99.96	100	100	100	100

**Table 8:** F1-Score.

F1-Score (%)	W0- > W1	W0- > W2	W0- > W3	W1- > W0	W1- > W2	W1- > W3	W2- > W0	W2- > W1	W2- > W3	W3- > W0	W3- > W1	W3- > W2
ShuffleNet	98.89	100	99.69	100	100	99.52	100	99.03	99.52	98.14	98.74	99.69
Squeezenet	100	100	99.51	100	99.03	100	100	100	92.21	100	100	100
MicroNet	100	100	100	100	100	100	100	100	99.72	100	100	100
MobileNetV3-Small	100	100	99.72	100	100	100	100	100	100	100	100	100
GhostNet	100	100	100	100	100	100	100	100	100	100	100	100
CNN	100	100	97.11	100	99.51	97.59	99.02	99.03	91.08	100	100	100
DAFNet	100	100	100	100	100	99.93	100	99.93	100	100	100	100

From the comprehensive analysis of Tables 6, 7 and 8 and Figs. 12, 13 and 14, it can be seen that, taking accuracy as an example, DAFNet exhibits excellent and stable performance in cross-condition recognition tasks. In all 12 transfer tasks, DAFNet achieves or approaches 100% accuracy in the vast majority of items, performing particularly well in multiple cross-domain scenarios with significant distribution differences such as W0- > W3, W1- > W3, and W2- > W3. Compared with other mainstream lightweight models, DAFNet’s performance is basically on par with the best-performing MicroNet, MobileNetV3-Small and GhostNet, while significantly outperforming other models; for instance, baseline CNN and SqueezeNet show obvious performance degradation in some transfer paths (especially W2- > W3, W0- > W3), dropping to as low as 92.46%, whereas DAFNet consistently remains at a high level without significant fluctuations. Looking at the test results of recall rate and F1-Score, DAFNet also maintains a comprehensive and consistent lead. This verifies that the high performance of DAFNet is not accidental, but an inevitable reflection of the model’s own strong generalization ability, which can effectively avoid misjudgment on a certain category and ensure the reliability of the diagnostic results. It is worth noting that, combined with Table 4, it can be seen that DAFNet achieved the aforementioned equivalent superior generalization performance using only about 1/8 of the parameter count of MicroNet, about 1/38 of that of MobileNetV3-Small, and about 1/91 of that of GhostNet, which further proves the efficiency of its architecture.

## Conclusion

Aiming at the problem that existing deep learning models often accompany a surge in parameter volume when pursuing feature extraction capability and are difficult to deploy on resource-constrained devices, this paper proposes a motor bearing fault diagnosis method based on Disentangle-and-Aggregate Feature Learning Network (DAFNet). This method directly takes raw one-dimensional vibration signals as input, and extensive comparative experiments and generalization performance verification were conducted on the CWRU dataset, mainly drawing the following conclusions:

1. By introducing the “disentangle-and-aggregate” mechanism, DAFNet changed the pattern of traditional CNNs improving performance purely by stacking layers, breaking the positive correlation contradiction between network depth and parameter volume. Experiments show that the network successfully avoided parameter redundancy while effectively utilizing shallow details, medium context, and deep semantic information.
2. It achieved extreme inference efficiency and low computational energy consumption, meeting industrial real-time requirements. In the efficiency comparison experiment, DAFNet showed an overwhelming advantage. Its parameter count is only about 42,000, computational complexity is as low as 0.1 MFlops, and inference speed reaches up to 8,755.51 FPS, which is 6.6 ~ 140 times that of other comparison networks. This indicates that the model greatly reduced the dependence on hardware computing power and can realize real-time fault diagnosis on embedded edge devices with extremely low energy consumption.
3. It broke through the performance bottleneck of lightweight models and achieved the optimal balance between accuracy and speed. Although existing methods on the CWRU dataset generally perform well, DAFNet still maintained an average diagnostic accuracy of 100% while compressing the model to the extreme, and showed generalization ability superior to or on par with other mainstream lightweight models in cross-condition experiments. This result confirms that DAFNet does not simply sacrifice accuracy for speed, but achieves complete expression of fault features under a minimalist architecture through an efficient feature fusion mechanism, providing an efficient and reliable theoretical basis and technical scheme for intelligent operation and maintenance of motor bearings in the context of Industrial Internet of Things.

## Data Availability

The datasets used and/or analysed during the current study available from the corresponding author on reasonable request.
